# Functionally Significant Variants in Genes Associated with Abdominal Obesity: A Review

**DOI:** 10.3390/jpm13030460

**Published:** 2023-03-01

**Authors:** Ahmad Bairqdar, Dinara Ivanoshchuk, Elena Shakhtshneider

**Affiliations:** 1 Federal Research Center Institute of Cytology and Genetics, Siberian Branch of Russian Academy of Sciences, Prospekt Akad. Lavrentyeva 10, Novosibirsk 630090, Russia; 2 Institute of Internal and Preventive Medicine—Branch of Institute of Cytology and Genetics, Siberian Branch of Russian Academy of Sciences, Bogatkova Str. 175/1, Novosibirsk 630004, Russia

**Keywords:** adipocyte, obesity, gene

## Abstract

The high prevalence of obesity and of its associated diseases is a major problem worldwide. Genetic predisposition and the influence of environmental factors contribute to the development of obesity. Changes in the structure and functional activity of genes encoding adipocytokines are involved in the predisposition to weight gain and obesity. In this review, variants in genes associated with adipocyte function are examined, as are variants in genes associated with metabolic aberrations and the accompanying disorders in visceral obesity.

## 1. Introduction

High prevalence of obesity and of its associated diseases is a major problem worldwide [1]. Genetic predisposition and an influence of environmental factors that contribute to the development of obesity represent a set of causes of excess body weight [2,3,4,5,6]. Hereditary factors involved in the development of obesity lead to the formation of syndromic, monogenic, and polygenic types of obesity [7]. 

Monogenic obesity is a pathology caused by a mutation in a single gene. Monogenic obesity is extremely rare and is characterized by early onset, development in childhood, and extreme values of body weight [8,9]. Monogenic types of obesity are caused by a mutation in one of genes *LEP*, *LEPR*, *POMC*, *PCSK1*, and *MC4R*, which encode proteins of the leptin–melanocortin system. This system is key for the regulation of eating behavior and energy metabolism [10]. The leptin–melanocortin system is activated by leptin, which is secreted by adipocytes but exerts its action via leptin receptor, thereby leading to the activation of pro-opiomelanocortin. Under the influence of an enzyme called prohormone convertase 1, adrenocorticotropic hormone and α-melanocyte-stimulating hormone are generated from proopiomelanocortin; α-melanocyte-stimulating hormone in turn activates receptor MC4R, which launches satiety signaling [8]. Environmental factors have little effect on the development of nonsyndromic monogenic obesity but may be crucial for treatment. Physical activity, socioeconomic status, and diet type may influence obesity severity in these patients [11].

Syndromic obesity is obesity that develops in chromosomal and other genetic syndromes (e.g., Prader–Willi, fragile X, Bardet–Biedl, Cohen, and Albright) and is accompanied by a set of congenital aberrations [11].

Polygenic obesity is caused simultaneously by many genes and their interactions both with each other and with the external environment. Polygenic obesity is widespread in many populations [12]. In research on polygenic etiology of obesity by means of genome-wide association studies, 127 loci associated with obesity have been found in the human genome [7]. This number constantly changes as new studies come out regarding the polygenic etiology of obesity as a complex multifactorial disease [13]. Although polygenic obesity is the most common type of obesity and is the most responsive to clinical interventions, it is the least investigated owing to its sensitivity to environmental factors and because of its variation among ethnic groups [14].

Obesity is associated with various pathological metabolic changes in the human body: impaired glucose tolerance, insulin resistance, dyslipidemia, nonalcoholic fatty liver disease, and hypertension [15,16,17,18,19]. Visceral adiposity may have a negative effect on longevity [20]. From the point of view of the development of metabolic disorders in obesity, it is important to study visceral adipose tissue and its role as a hormonally active structure [21]. Adipocytes of visceral adipose tissue secrete a set of biomolecules: adipocytokines, which are signaling and regulatory peptides that regulate various metabolic processes [22,23,24]. In Whites with a BMI ≥ 25.0 kg/m^2^ and in Asians with a BMI ≥ 23.0 kg/m^2^, waist circumference (WC) measurement is recommended as a simple and informative method for diagnosing abdominal obesity. Values of WC ≥ 80 cm in women and WC ≥ 94 cm in men are said to correspond to abdominal obesity and increased risk of cardiovascular events [25,26]. This method does not apply to pregnant and lactating women.

Changes in the structure and function of genes encoding adipocytokines are involved in the predisposition to weight gain and obesity [7,27,28]. In this review, selected genes associated with adipocyte function are examined, as are variants in genes associated with metabolic aberrations that contribute to visceral obesity (Table 1). 

In this review, we analyzed the PubMed database [48] by means of the following search string: “abdominal obesity” [Title/Abstract] AND “single nucleotide polymorphism” [Title/Abstract]. We found 177 full-text articles for the period 2002–2022. Only 20 of the 177 articles (11.3%) contained information on polymorphic variants of genes associated with abdominal obesity. These 20 articles were included in the review.

**Figure 1:**
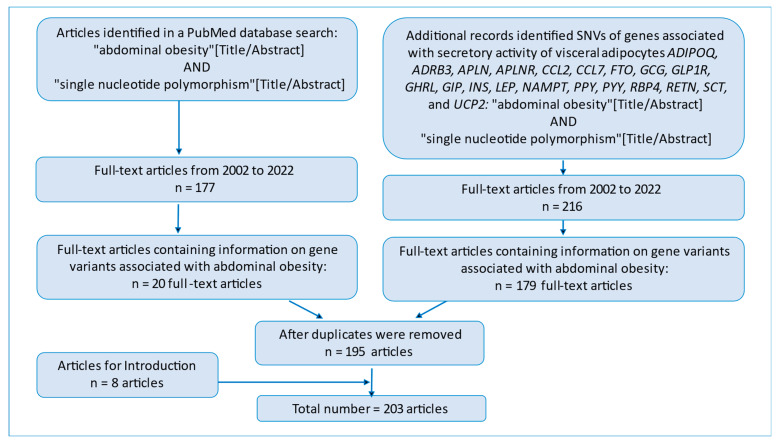
A flowchart of the initial publications in PubMed.

At the next stage, we performed an additional search in PubMed for articles containing information about single-nucleotide polymorphisms in genes associated with adipocyte secretory activity: *ADIPOQ*, *ADRB3*, *APLN*, *APLNR*, *CCL2*, *CCL7*, *FTO*, *GCG*, *GLP1R*, *GHRL*, *GIP*, *INS*, *LEP*, *NAMPT*, *PPY*, *PYY*, *RBP4*, *RETN*, *SCT*, and *UCP2*. At this stage, we included 175 full-text articles in the review (Figure 1).

We included an additional 8 full-text articles in Introduction. In this review, the total number of articles is 203.

Variants in the genes examined in our review participate in the development of polygenic obesity.

## 2. Results

### 2.1. ADIPOQ

The *ADIPOQ* gene encodes the protein adiponectin, which shares similarities with collagens X and VIII and complement factor C1q. The gene is expressed predominantly in adipose tissue, and the encoded protein circulates as various isoforms in blood plasma. Adiponectin participates in the regulation of many metabolic and hormonal processes, including carbohydrate and lipid metabolism, and has anti-inflammatory effects. Adiponectin interacts with two types of receptors: AdipoR1 and AdipoR2 [49]. Mutations in this gene can lead to adiponectin deficiency [50]. Adiponectin levels inversely correlate with both total body weight and visceral fat mass [51].

The *ADIPOQ* gene has many variants that result in low adiponectin levels, which correlate with obesity: the rs1391272583 variant in a Brazilian population [52], rs17366568 in a Malaysian population [53], rs266729 in a young Nigerian population [54], variants rs266729, rs16861205, rs1501299, rs3821799, and rs6773957 in a Finnish population [55], and variants rs17846866 and rs1501299 in the Indian population of Gujarat [56]. Other variants in this gene have been associated with type 2 diabetes mellitus (T2DM): rs62625753 and rs17366743 in a French white population [57], rs185847354 in a Japanese population [58], and rs2241766, rs2082940, and rs266729 in a Finnish population [55]. The *ADIPOQ* rs266729-G allele affects body fatness in response to dietary monounsaturated fatty acids [59]. 

### 2.2. ADRB3

This gene encodes a receptor that belongs to the β-adrenergic receptor family. The activity of the gene is regulated by catecholamines. ADRB3 is expressed mostly in adipose tissue and is responsible for the modulation of lipolysis and thermogenesis [60]. Lipolysis-produced free fatty acids via upregulation of *ADRB3* accelerate transcription of the *UCP1* gene and raise the activity of the UCP1 protein [61].

Several variants of the *ADRB3* gene are associated with the development of obesity and T2DM. One of the first studied variants in this gene, rs4994, has been found to correlate with severe cases of obesity and insulin resistance in a Japanese population [62]. Subsequently, the rs4994 variant has been investigated in many populations and has shown an association with the risk of childhood and adolescent obesity and being overweight [63]. The correlation of rs4994 (*ADRB3* gene) with obesity may be mediated by the impact of this polymorphism on adipokines. Carriers of its C allele have anomalous blood levels of adipokines and lipids [64]. Variants rs72655364 and rs72655365 of this gene correlate with T2DM in a Chinese population [65].

### 2.3. APLN and APLNR

The *APLN* gene codes for a preproprotein that is subsequently cleaved and activated in the endoplasmic reticulum. The protein apelin is secreted as a peptide hormone that binds to apelin receptors located in various organs. Apelin plays an important part in the regulation of many biological functions, including insulin secretion [66].

The rs2281068 variant (*APLN* gene) is associated with T2DM in the Chinese Han population [67], whereas the rs3115757 variant correlates with obesity among women in a Chinese population [68]. The receptor (encoded by the *APLNR* gene) to which apelin binds is expressed in the spleen, brain, placenta, and adipose tissues [69].

Studies on *APLNR* gene variants have not detected any associations with diabetes mellitus or obesity [70]. Some variants in the *APLNR* gene correlate with the risk of hypertension [71].

### 2.4. CCL2 and CCL7

Genes *CCL2* and *CCL7* are members of the CC subfamily of the chemokine superfamily, which plays a crucial role in immunomodulatory and inflammatory processes. The two genes are located side by side on chromosome 17 [72].

The serum concentration of CCL2 correlates with insulin resistance and a high body–mass index (BMI) [73]. In a Mexican population, the G allele of variant rs1024611 is associated with lower serum insulin levels, a lower BMI, lower adipose-tissue volume, and higher adiponectin levels than is the A allele [74]. The G allele of variant rs1024611 is less common among women with gestational diabetes mellitus, whereas its GG genotype is associated with a lower BMI among women with gestational diabetes mellitus [75].

CCL7 is overexpressed in the adipose tissue of obese people [76]. This upregulation is induced by elevated expression of interferon regulatory factor 5 in adipose tissue [77]. 

### 2.5. FTO

The *FTO* gene codes for a protein that plays an important part in the development of obesity and T2DM [78]. The *FTO* gene was one of the first identified loci correlating with obesity in genome-wide association studies [7]. This gene is widely expressed within the human body, with the highest expression in the brain. Several reports from different populations indicate an association of some variants of *FTO* with obesity in children and adults [79]. Two of the earliest genome-wide association studies in this field showed a correlation of *FTO* variants with early onset of obesity in a German population [80] and with T2DM in a Finnish population [81]. The rs1421085 variant of *FTO* is associated with impaired differentiation of adipocytes. The *FTO* SNP rs1421085 disrupts the binding site of repressor ARID5B [33]. The *FTO* SNP rs1558902 correlates with the BMI [82,83]. *FTO* variants implicated in obesity have been found in diverse populations: rs1421085, rs8050136, and rs9939609 in an Indian population [84], rs9939609 in a Russian population [85], rs9939609, rs1121980, and rs1558902 in a Japanese population [86,87], and rs9939609 in a Saudi Arabian population [88]. Rs9939609 is associated of with the risk of central obesity in Chinese children [89]. Rs9939609 has shown a significant correlation with male visceral obesity in an Indonesian population [90]. Increased abdominal fatness is associated with the AA genotype of a common SNP of *FTO* (rs9939609, T/A) when measured as waist circumference and intra-abdominal adipose tissue [91]. On the other hand, the rs9939609 polymorphism of this gene correlates with fat accumulation in the whole body without being associated with abdominal fat accumulation in Turkish adults [92]. Rs9939609 is in the same linkage region as rs8050136 (r2 = 1) [93]. A rare variant—rs140101381 (R80W)—is associated with early onset of obesity [27]. The T allele at the rs3751812 locus has been implicated in increased waist circumference in Asian-Indian populations [94]. Rs7185735 is associated with an increase in subcutaneous adipose tissue and a decrease in the visceral adipose tissue/subcutaneous adipose tissue ratio and correlates with the childhood BMI [95,96]. *FTO* rs9936385 is responsible for significant differences in the BMI between adults ≤50 years of age and adults aged >50 years [97].

### 2.6. GCG and GLP1R

The *GCG* gene encodes glucagon, a hormone that regulates blood glucose levels. GCG is expressed mainly in pancreatic α-cells in the islets of Langerhans and in the small intestine. As a consequence of a post-translational modification in α-cells of the pancreas, glucagon is generated, and in the small intestine, a post-translational modification results in the production of glucagon-like peptides 1 and 2, oxyntomodulin, and glicentin. Glicentin is a 69 amino acid peptide, which contains the entire sequences of oxyntomodulin (and hence glucagon) and glicentin-related pancreatic peptide [98].

*GCG* variants related to T2DM and obesity have been identified. In a Danish population, carriers of the homozygous GG genotype of rs4664447 have lower plasma glucose-stimulated insulin levels [99]. The rs12104705 variant correlates with general and abdominal obesity [34].

The *GLP1R* gene encodes a transmembrane receptor for glucagon-like peptide 1 [100]. The receptor is internalized by binding to GLP1 or its analogs, and activation of this receptor results in insulin secretion [35].

In 2004, an association of rs367543060 of the *GLP1R* gene with both impaired insulin secretion and aberrant insulin sensitivity was identified for the first time in patients with T2DM [101]. Variants rs2268641 and rs6923761 in this gene correlate with a high BMI [102,103]. Carriers of the AA genotype of rs6923761 have a higher risk of obesity and higher glucose levels [104]. Variant rs10305492 in a European population [105] and rs3765467 and rs10305492 in a Chinese population [106] have been implicated in metabolic syndrome and a higher risk of T2DM. Interaction between various factors, including the single-nucleotide polymorphisms (SNPs) that alter signaling, transport, and receptor activity, is key to the design of next-generation personalized agonists of GLP1R [107].

### 2.7. GHRL

The *GHRL* gene (ghrelin and obestatin prepropeptide) encodes a preproprotein that is later cleaved thereby yielding ghrelin and obestatin, which are expressed and secreted primarily in the stomach and much less often in the small intestine. The peptide ghrelin binds to its receptor (GHSR) in the hypothalamus and drives growth hormone secretion [108]. Ghrelin modulates many metabolic pathways and performs a crucial function in the reward system via the mesolimbic pathway [109]. Higher prevalence of variants rs34911341 and rs696217 has been found among obese people than in the general population [110,111].

Subsequent research has uncovered an association of various other *GHRL* variants with obesity: rs4684677 in a general-population cohort of European origin [112]; rs35682 and rs35683 in a white American population [113], and rs696217 in a Japanese population [114]; in addition, rs35681 correlates with the development of obesity in polycystic ovary syndrome [115].

Some variants in the *GHRL* gene are associated with disorders of carbohydrate metabolism. Rs27647 correlates with lower insulin levels in the oral glucose tolerance test (at 2 h after the glucose challenge) [112]. One of ghrelin’s effects related to weight changes is an alteration of eating behavior; for example, variants rs696217 and rs2075356 have been implicated in bulimia nervosa [116]. 

### 2.8. GIP

The *GIP* gene (gastric inhibitory polypeptide) is located on chromosome 17 and encodes an incretin hormone that regulates insulin secretion and ensures blood glucose homeostasis [117].

Three SNPs (rs3895874, rs3848460, and rs937301) have been analyzed in the 5′ region of the human *GIP* gene. Functional studies have revealed that in the promoter region of GIP, rare alleles of these three SNPs [haplotype GIP(−1920A)] correspond to significantly lower transcriptional activity than do the common alleles of these SNPs [haplotype GIP(−1920G)] [118]. Rs9904288, which is located at the 3′ end of *GIP*, is significantly associated with visceral fat area [119].

### 2.9. INS

The *INS* gene codes for the hormone insulin, which is responsible for the modulation of carbohydrate and lipid metabolism. The gene is located on chromosome 11 [120]. Insulin synthesis in pancreatic β-cells starts with preproinsulin [121]. During transport to the endoplasmic reticulum, the signal peptide located at the N terminus is cleaved off, then the remaining polypeptide is folded with the formation of disulfide bridges between the α and β chains, after which proinsulin is transported to the Golgi apparatus, where it is packaged into vesicles. Proinsulin is cleaved in vesicles and loses its C-peptide, which is subsequently secreted along with insulin molecules in equal amounts [122,123].

Some mutations in the insulin gene can lead to specific subtypes of diabetes, such as mutant *INS* gene–induced diabetes of youth (MIDY), maturity onset diabetes of the young (MODY), or neonatal diabetes [124,125]. Variants A24D, F48C, and R89C (*INS* gene), which are implicated in the development of neonatal diabetes, result in inefficient processing of proinsulin [126]. Rare variants R6H and R46Q have been described in familial cases of maturity onset diabetes of the young [127]. The A24D variant is associated with inefficient cleavage of preproinsulin [124], whereas the V92L variant weakens the affinity of insulin for insulin receptor [125], and both lead to mutant *INS* gene–induced diabetes of youth.

### 2.10. LEP

The leptin gene encodes a protein that is expressed by white adipocytes and secreted into the blood. Leptin plays an important role in the regulation of energy homeostasis and body weight control [128]. Leptin circulating in the blood binds to leptin receptor and has a central effect and peripheral effect. In the brain, leptin activates appetite-regulating signaling pathways that cause a decrease in food intake. The influence of metabolic stressors on leptin secretion has become a major focus of research on feeding behaviors [129]. The identification of central actions of leptin during signaling through its specific LepRls in neurons of neuroendocrine and hippocampal circuits has revealed complex integrated central control of body energy (expenditure vs. storage) and sophisticated self-defense of the brain, thus shedding light on how leptin modulates satiety and compulsion [130]. Reductions in energy reserves and in leptin production appear to cause a compensatory change in the reward system and the secretion of orexigenic and anorexigenic neuropeptides [131,132].

Leptin also regulates bone mass and the secretion of hypothalamic–pituitary–adrenal hormones. At the periphery, leptin accelerates basal metabolism and modulates the function of pancreatic β-cells and insulin secretion. In intestines, leptin activates protein kinase C and reduces glucose absorption [133]. Leptin levels also correlate with the waist-to-hip ratio and BMI. Leptin levels are higher in individuals with obesity compared to those without obesity [134].

Mutations in this gene may be the cause of severe obesity [133]. Mutations in the LEP gene have also been associated with the development of T2DM [128].

Mutations in the leptin gene leading to leptin deficiency are some of the causes of monogenic obesity with such symptoms as impaired satiety, hyperphagia, early onset of obesity, and many metabolic and immunological disorders [135]. These autosomal recessive pathogenic mutations are well studied and differ from the LEP variants that do not lead to monogenic obesity but may be a risk factor of obesity and other metabolic disorders.

In a study on common variants in the leptin gene, researchers showed an association of the AA genotype of the rs7799039 variant with the development of obesity in the population of South India [136]. The A allele of rs2167270 significantly correlates with an elevated risk of prediabetes in Jordan and in the population of South India [137,138,139]. Rs6966536 (allele G) of the LEP gene has been implicated in the development of obesity in a South African population [140]. 

Among African Americans, two SNPs of the LEP gene (rs4731427 and rs17151919) have been associated with weight, BMI, and WC. Among whites, rs2167270 and rs17151913 (in this gene) correlate with weight, whereas in women, rs28954369 is associated with weight, BMI, and WC [141]. In a study by Yaghootkar et al., missense variant Val94Met (rs17151919) was found to occur only among people of African descent, and its association with lower concentrations of leptin was specific to this origin [142].

Despite abundant research on leptin, the mechanisms of action of variants on the expression and stability of leptin or on its interaction with its receptor are still being investigated. A study by Hagglund et al. offers an explanation that includes the following mechanisms: (1) weaker affinity of leptin for its receptor, (2) blockade of leptin receptor, (3) destabilization of leptin, and (4) improper coagulation/aggregation of leptin [143].

### 2.11. NAMPT

This gene encodes an enzyme that catalyzes the condensation of nicotinamide with 5-phosphoribosyl-1-pyrophosphate. The gene is located on chromosome 7 [144]. The enzyme is secreted into extracellular space, and its secreted form is known as visfatin. The latter acts as a cytokine and adipokine [145].

Many *NAMPT* variants have been identified that correlate with changes in its metabolic function and adipokine activity. The T allele of the −948G/T variant is associated with lower fasting insulin levels [146] and higher levels of high-density lipoprotein cholesterol [147]. *NAMPT* variants rs10487818 and rs3801266 correlate with changes in body weight [148,149]. The rs3801267 variant has shown an association with a lower BMI in a Chinese population [150]. Rs34861192 and rs13237989 (*NAMPT*) affect insulin levels and the glycemic index [151]. The aforementioned variants cause either higher or lower serum levels of visfatin, which in turn leads to alterations of insulin and blood glucose levels [152].

### 2.12. PYY and PPY

Genes *PPY* and *PYY* code for two peptides from the neuropeptide family: pancreatic polypeptide (PP) and peptide YY (PYY). Both genes are located on chromosome 17 [153,154].

Neuropeptides typically affect the gut–brain axis by regulating appetite and energy homeostasis. The peptides of this family act through five receptors called “Y-receptors”: Y1, Y2, Y4, Y5, and Y6. These receptors differ in their tissue distribution, function, and selectivity of binding to various neuropeptides. Peptide PYY acts on the arcuate nucleus of the hypothalamus, where the Y-receptors are located that have strong affinity for PYY [155]. Gene variants in *PYY* are associated with changes in body weight and with obesity. Variants rs11684664, rs162430, and rs1058046 have been implicated in the development of obesity in various reports [156,157,158].

Peptide PPY is synthesized in the pancreas as a 94-amino-acid polypeptide; then, it is cleaved into 2 peptides, with only the 36-amino-acid PPY fragment being active [153]. The secreted peptide performs a function similar to that of PYY but has stronger affinity for receptor Y4. The PPY peptide is secreted by very rare γ-cells in pancreatic islets [159]. According to one research article, the GG genotype of variant rs231472 in the *PPY* gene correlates with the risk of obesity in children in Korea [160].

### 2.13. RBP4

The *RBP4* gene is located on chromosome 10 and codes for a protein that binds retinol and helps to transport it from the skin to peripheral tissues. The protein is expressed mostly in the liver (and at lower levels in adipose tissues). Retinol bound to RBP4 is then imported into the cell by STRA6, which serves as a receptor of the RBP4–retinol complex [161].

RBP4 was first described as an adipokine in 2005 [162]. Variants rs34571439 and rs3758539 are associated with decreased insulin secretion in whites [163]. The rs34571439 variant (*RBP4* gene) has been implicated in hypertriglyceridemia [164] and childhood obesity [165]. Variant rs7091052 is associated with the risk of gestational diabetes mellitus [166].

### 2.14. RETN

This gene encodes a protein called resistin. The resistin gene is located on chromosome 19 [167]. Resistin has a pleiotropic role in inflammation, in the biology of stress, and in the pathogenesis of obesity [168].

Variants in the *RETN* gene correlate with signs of metabolic syndrome. Investigation has revealed an association of variant rs1862513 with the risk of obesity and T2DM [169,170]. Other variants (rs3745367, rs1423096, and rs10401670) of this gene have been associated with some parameters of metabolic syndrome in various populations [171,172,173].

Differences in serum resistin levels may be related to alterations of methylation patterns in the RETN promoter region [174]. Variants in coding regions of RETN have a greater impact on this protein’s stability [175].

### 2.15. SCT

The *SCT* gene is located on chromosome 11 and codes for hormone secretin. Secretin stimulates bile and bicarbonate secretion in the duodenum, pancreas, and bile ducts [176]. Secretin expression activates brown adipose tissue, reduces central responses to appetizing food, and delays motivation for eating again after a meal [177,178].

### 2.16. UCP2

The *UCP2* gene is in the same cluster as the *UCP3* gene and is situated on chromosome 11 [179]. It is thought to take part in unregulated thermogenesis, obesity, and diabetes mellitus [180]. Variant rs659366 is associated with the risk of obesity [85,181,182,183,184], whereas variant rs660339 correlates with the risk of obesity in a specific population: Indonesians [185,186,187]. 

Modern molecular genetic and biochemical technologies are key to solving such fundamental problems as the formation of overweightness and obesity, thereby making it possible to identify genes and their products participating in the pathogenesis of obesity, to investigate the molecular mechanisms of relevant pathological processes, and to determine molecular biological heterogeneity of pathological phenotypes [7,188].

In addition to the determination of the BMI and WC, a Body Shape Index (ABSI) is used to diagnose obesity. ABSI takes into account body height, weight, and WC. ABSI has independent genetic associations [189,190]. ABSI can more precisely quantify abdominal obesity in metabolic syndrome [191]. Other indicators may also help to diagnose abdominal obesity, but all of them maintain a significant correlation with the BMI [192]. The association of polymorphisms of genes with ABSI may be an interesting research topic for clarifying functional significance of SNPs.

An effective method of early diagnosis and prevention of obesity is the identification of genetic markers of predisposition to this pathology and research on their characteristics in various populations [193]. Most results presented in the review have been obtained in case-control studies. This method complements data from genome-wide association studies [194,195,196]. Table 2 shows the main variants that are associated with metabolic disorders and located in the genes presented in this review.

Based on the analysis of functional significance of the variants, a scale of an individual’s genetic risk of obesity may be constructed. The creation of such a scale is an important task for modern endocrinology. The complexity of this task is due to population specificity of (i) prevalence rates of obesity, (ii) characteristics of climatic and social living conditions, and (iii) genetic factors. When a scale of individual genetic risk is created, it is important to take into account the ethnicity of the patients included in the research. For example, according to many studies, the same scale of individual genetic risk detects an association with cardiovascular events in one population and does not in another [197,198]. At present, there are not many published studies on the development of an ethnospecific scale of individual genetic risk of obesity. Most of these studies have been conducted on white cohorts, and the results may not be applicable to other populations. In research projects examining the association between a scale of individual genetic risk and diseases, Cox’s proportional hazards model and logistic regression analysis are used. Correlations between a genetic risk scale and anthropometric and biochemical parameters are assessed via linear regression. ROC (receiver operating characteristic), the area under the ROC curve (i.e., C-statistic), NRI, and integrated discrimination improvement are metrics of the quality of a scale of individual genetic risk as compared to traditional risk factors [199].

The risk of obesity, as assessed by the genetic risk scale, can be modified, for example, by a therapy aimed at weight loss and alleviation of comorbidities. It is now recognized that our everyday exercise and nutrition choices have long-term consequences for our brain function [200,201,202,203]. Early detection of a genetic risk may help to improve quality of life and life expectancy and to reduce economic costs of treatment.

## 3. Conclusions

It is necessary to study each population, both in terms of the nature of the variation of genes predisposing to diseases associated with excess body weight and in terms of specific features of their phenotypic manifestation. High-throughput sequencing technologies allow investigators to obtain new information about the variation of the structure of genes in groups from the general population and in clinical groups of overweight and obese individuals.

## Figures and Tables

**Table 1:** Genes examined in this review that are associated with secretory activity of visceral adipocytes.

Gene	Genes Associated with the Secretory Activity of Visceral Adipocytes	References
*ADIPOQ*	This gene is expressed in adipose tissue exclusively. Mutations in this gene are associated with adiponectin deficiency.	[29]
*ADRB3*	This receptor is located mainly in adipose tissue and is involved in the regulation of lipolysis and thermogenesis. Obesity-related and body weight–related disorders correlate with polymorphisms in this gene.	[30]
*APLN*, *APLNR*	Apelin is secreted by adipose tissue. Apelin and its receptor are widespread in the human body and take part in many physiological processes, such as glucose and lipid metabolism, homeostasis, endocrine responses to stress, and angiogenesis.	[31]
*CCL2*, *CCL7*	Chemokines CCL2 and CL7 have been implicated in the pathogenesis of several disorders, including obesity.	[32]
*FTO*	This gene shows a strong association with the BMI, obesity risk, and T2DM.	[33]
*GCG*	The glucagon (GCG) family of peptide hormones plays a role in central control of feeding behavior.	[34]
*GLP1R*	The hormone called glucagon-like peptide 1 (GLP-1) plays an important part in the signaling cascades resulting in insulin secretion.	[35]
*GHRL*	This gene encodes ghrelin-obestatin preproprotein, which is cleaved thus yielding two peptides: ghrelin and obestatin. Ghrelin regulates multiple phenomena, including hunger and pancreatic glucose-stimulated insulin secretion. Obestatin has multiple metabolic functions, including regulation of adipocyte function and glucose metabolism.	[36]
*GIP*	This gene encodes an incretin hormone. This protein is important for glucose homeostasis because it is a potent stimulator of insulin secretion from pancreatic β -cells after food ingestion and nutrient absorption.	[37]
*INS*	This gene codes for insulin: a peptide hormone that plays a vital role in the regulation of carbohydrate and lipid metabolism.	[38]
*LEP*	This gene encodes a protein that is secreted by white adipocytes into the circulation and performs a major function in the modulation of energy homeostasis. Circulating leptin binds to leptin receptor in the brain, thereby triggering downstream signaling pathways that inhibit feeding and promote energy expenditure. Mutations in this gene and in its regulatory regions induce severe obesity.	[39]
*NAMPT*	This gene encodes a protein that participates in many important biological processes, including metabolism, stress responses, and aging. Levels of NAMPT in adipose tissue are rather high.	[40,41]
*PPY*	This hormone acts as a regulator of pancreatic and gastrointestinal functions and may be important for the modulation of food intake.	[42]
*PYY*	Rare variations in this gene may increase susceptibility to obesity.	[42]
*RBP4*	Vitamin A can affect obesity progression and the development of obesity-related diseases including insulin resistance, T2DM, hepatic steatosis, steatohepatitis, and cardiovascular diseases.	[43]
*RETN*	This gene codes for a protein called resistin. Resistin is secreted by adipocytes and may be the hormone potentially linking obesity to T2DM.	[44,45]
*SCT*	Secretin activates brown adipose tissue, reduces central responses to appetizing food, and delays the motivation to refeed after a meal.	[46]
*UCP2*	This gene is expressed in many tissues, and the highest expression is seen in skeletal muscle. The product of this gene plays certain roles in unregulated thermogenesis, obesity, and diabetes mellitus.	[47]

**Table 2:** Single-nucleotide variants associated with metabolic disorders.

Gene	dbSNP ID	Nucleotide Changes	Type of Variation/Amino Acid Changes	Minor Allele Frequency (GnomAD)	ClinVar Variation ID/LOVD Database ID	Associated Metabolic Disorder
*ADIPOQ*	rs62625753	+268 G > A	Missense Variant G > S	0.004625	708724	allele A is associated with T2DM risk
*ADIPOQ*	rs266729	C > G	2KB Upstream Variant	G = 0.09786	-	allele C is associated with T2DM risk
*ADIPOQ*	rs17366743	+331 T > C	Missense Variant Y > H	0.030445	-	allele C is associated with T2DM risk
*FTO*	rs9939609	46-23525 T > A	Intron Variant	0.41025	-	allele A is associated with obesity
*FTO*	rs1421085	46-43098 T > C	Intron Variant	0.419704	214481	allele C is associated with obesity
*GCG*	rs4664447	254 + 672 A > T	Intron Variant	C = 0.00792 G = 0.00000	-	GG genotype is associated with insulin level abnormalities
*GHRL*	rs34911341	152 G > A	Missense Variant R > Q	0.007960	20100/ 00293181	allele A is associated with metabolic syndrome
*GHRL*	rs696217	214 C > A	Missense Variant L > M	0.080526	20101	allele T is associated with metabolic syndrome and obesity
*GHRL*	rs4684677	269 A > T	Missense Variant Q > L	0.06097	20102	allele A is associated with obesity
*GLP1R*	rs2268641	1224 + 1751 C > T	Intron Variant	0.39129	-	Associated with BMI
*GLP1R*	rs6923761	502 G > A	Missense Variant G > S	0.324795	-/00208599	GG genotype is associated with higher BMI and CV risks
*GLP1R*	rs10305492	946G > A	Missense Variant A > T	0.015874	-	allele A is associated with T2DM risk
*NAMPT*	rs9770242	C > A	2KB Upstream Variant	C = 0.23919	-	allele C is associated with lower fasting plasma glucose and insulin levels
*NAMPT*	rs1319501	T > C	2KB Upstream Variant	C = 0.23662 A = 0.00001	-	allele C is associated with lower fasting plasma glucose and insulin levels
*NAMPT*	rs10487818	448-303 T > A	Intron Variant	0.01841	-/00028958	allele T is associated with protection against obesity
*PYY*	rs11684664	C > T		A = 0.0000 T = 0.00038		allele T is associated with obesity-related phenotypes in women only
*PYY*	rs162430	270-61 C > T	Intron Variant	0.105072	-	allele A is associated with childhood obesity
*RBP4*	rs34571439	A > C	500B Downstream Variant	0.186457	-	allele C is associated with reduced insulin secretion
*RBP4*	rs3758539	C > T	2KBUpstream Variant	0.16303	-	allele T is associated with reduced insulin secretion
*RETN*	rs3745367	118 + 181 G > A	Intron Variant	0.248110	-	allele A is associated with obesity
*UCP2*	rs659366	C > T	2KB Upstream Variant	0.370248	22904	allele T is associated with higher BMI
*UCP2*	rs660339	164 C > T	Missense Variant A > V	0.402632	136143	allele A is associated with obesity

## Data Availability

Not applicable.
